# Clinical, Radiological, and Genetic Profiles of Eight Patients with Combined Dystonic Manifestation of Type-III GM1 Gangliosidosis: A Video Case Series from India

**DOI:** 10.5334/tohm.1152

**Published:** 2026-02-09

**Authors:** Subhajit Roy, Cheshta Arora, Vikram V. Holla, Shweta Prasad, Prashant Phulpagar, Nitish Kamble, Babylakshmi Muthusamy, Jitender Saini, Ravi Yadav, Pramod Kumar Pal

**Affiliations:** 1 National Institute of Mental Health and Neuro Sciences (NIMHANS), Bangalore, India; 2 Institute of Bioinformatics, International Technology Park, Bangalore, India; 3 Manipal Academy of Higher Education, Manipal, India

**Keywords:** GM1, Gangliosidosis, *GLB1*, Dystonia, Parkinsonism, Wishbone

## Abstract

**Background:**

Type-III (adult/chronic) GM1 gangliosidosis is an uncommon, late-onset lysosomal disorder that frequently presents as a complex movement disorder.

**Methods:**

In this retrospective case series, clinical details, neuroimaging, electrophysiology, and genetics were extracted from standardized records and videos.

**Results:**

Eight patients were identified with median age at symptom onset of 6 years (range 3–18), and age at presentation of 23 years (12–27). All exhibited generalized dystonia with early, conspicuous oro-mandibular-cranio-cervical involvement; dysarthria was universal, parkinsonism occurred in two, and corticospinal signs in six. Ocular motor abnormalities were frequent; kyphoscoliosis was common. Where performed, nerve conduction studies, electroencephalography, evoked potentials, and abdominal ultrasound were unremarkable. MRI consistently demonstrated bilateral posterior putaminal T2/FLAIR change and the pathognomonic pallidal SWI “wishbone” pattern. All patients harboured biallelic *GLB1* variants: seven were compound heterozygous and one was homozygous. The recurrent variant c.1325G>A;p.Arg442Gln was present in seven patients. One novel variant (c.1022G>T;p.Gly341Val) was identified. Symptomatic therapies yielded variable, generally modest benefits over available follow-up.

**Discussion:**

A prominent oromandibular–cranio–cervical dystonia, posterior putaminal atrophy, and hyperintensity, with a SWI “wishbone” sign, strongly point to Type-III GM1 gangliosidosis. Recognizing this clinico-radiologic–genetic constellation can streamline targeted testing and counselling.

## Introduction

GM1 gangliosidosis is an uncommon autosomal recessive lysosomal storage disorder resulting from biallelic pathogenic variants in the *GLB1* gene, which encodes the lysosomal enzyme β-galactosidase. This enzyme is essential for degrading GM1 gangliosides and related glycoconjugates. Its deficiency leads to progressive intracellular accumulation, particularly in neurons, causing widespread neurodegeneration. Based on the clinical phenotype, GM1 gangliosidosis has been classified into three types: type-I or infantile form with onset between birth and 6 months, rapidly progressive with hypotonia, severe central nervous system (CNS) degeneration and death by 1–2 years of age; type-II including late infantile or juvenile form with onset usually between 7 months and 3 years, lag in motor and cognitive development, and slower progression; and type-III or adult/chronic variant with later onset between 3 to 30 years, a progressive movement disorder [1].

Pathogenic variants in the *GLB1* gene on chromosome 3p21.33 produce different phenotypes depending on the region affected. Mutations in exons 2, 6, 15, and 16 are typically associated with neurodegenerative features of GM1 gangliosidosis, while mutations near the 3’ end result in Morquio B disease, which mainly affects the skeleton [2]. Variants in the core protein region tend to result in early infantile presentations, whereas those affecting surface regions manifest later (types II and III).

Patients with Type-III GM1 gangliosidosis typically retain 5–10% of enzyme activity [1]. Juvenile and adult-onset forms often present with complex movement disorders. Patients typically develop generalized dystonia with prominent oromandibular, lingual, and facial involvement, along with slurred or spastic speech, spasticity, and progressive gait difficulties. Cognitive dysfunction may vary and is not universally present in early stages. A combination of progressive dystonia involving bulbar and appendicular muscles, along with corticospinal signs, can serve as key diagnostic indicators [3].

The adoption of next-generation sequencing (NGS) has enhanced diagnostic precision, particularly in cases of atypical or late-onset presentations. However, underdiagnosis remains common in developing nations due to limited awareness and constrained access to molecular testing. In this case series, we describe eight genetically confirmed patients with Type-III GM1 gangliosidosis from a tertiary care center in India. We aim to delineate the clinical and imaging features, highlight recurrent genetic variants, and emphasize the importance of considering GM1 gangliosidosis in young-onset, cranial-predominant, generalized, combined dystonia.

## Methods

This is a retrospective, descriptive case series conducted at the Department of Neurology, National Institute of Mental Health and Neurosciences (NIMHANS), India. The study included patients with genetically confirmed GM1 gangliosidosis, managed by the authors, between January 2019 and May 2025. The available demographics, clinical history, examination details, and investigations, including imaging, electrophysiological, and genetic data, were documented. Additionally, patient videos were reviewed when available. The data were expressed using descriptive statistics. The waiver was obtained from the National Institute of Mental Health and Neurosciences Institute Ethics Committee (No. NIMH/DO/IEC (BS & NS DIV)/2024-25) as the study involved retrospective data and the data were anonymised. Written informed consent was taken from the patients for the video recording and for publication in print or online.

## Results

**Figure 1:**
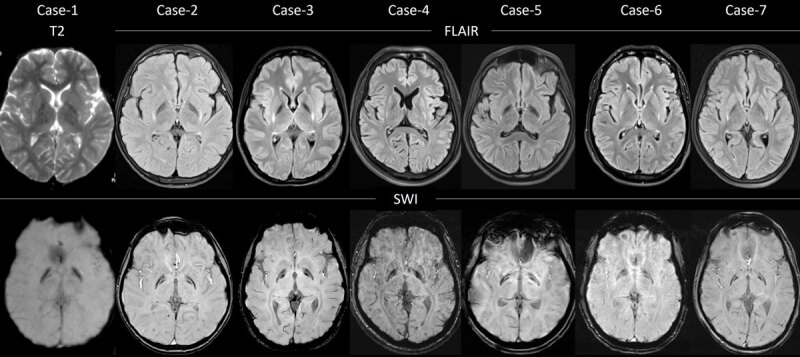
**Magnetic resonance imaging of the brain**. Axial T2, FLAIR (upper panel) and SWI sequences (Lower panel) of MRI brain of all the seven patients at the level of basal ganglia demonstrating bilateral symmetrical posterior putaminal atrophy and T2/FLAIR hyperintensity (upper panel) and mineralization of bilateral globus pallidi interna in the “wishbone” pattern on the SWI sequence (Lower panel). FLAIR: Fluid attenuated inversion recovery; MRI: Magnetic resonance imaging; SWI: susceptibility weighted imaging.

Eight patients (6 males) with genetically confirmed GM1 gangliosidosis were identified and recruited. The clinical, investigations, treatment, and follow-up details of all eight patients are summarized in Table 1. MRI imaging findings of seven patients are provided in Figure 1.

**Table 1:** Demographic, clinical features, investigation findings and follow-up details of the cohort.


VARIABLES	CASE 1	CASE 2	CASE 3	CASE 4	CASE 5	CASE 6	CASE 7	CASE 8

***Demographics***								

Age/AAO/Gender	21y/5y/M	16y/3y/M	23y/6y/F	27y/3y/M	24y/18y/F	24y/12y/M	12y/6y/M	21y/11y/M

FH/Consanguinity	+/–	+/–	–/–	+/–	–/–	+/–	–/–	–/+

***Symptoms***								

Presenting symptoms	Walking difficulty, slurring of speech, generalized abnormal posturing	Walking difficulty, slurring of speech, generalized abnormal posturing	Generalized abnormal posturing, slurring of speech, walking difficulty	Walking difficult, slurring of speech with generalized abnormal posturing	Walking difficulty, slurring of speech, generalized abnormal posturing	Walking difficulty, slurring of speech with generalized abnormal posturing	Generalized abnormal posturing, slurring of speech, walking difficulty	Walking difficulty, Slurring of speech, abnormal posturing

DD/ID	–/+	–/+	–/-	–/-	+/–	–/+	–/–	–/+

***Examination findings***								

Eye movements	Range, saccades, pursuit normal	Range, saccades, pursuit normal	Mild vertical gaze impairment	Mild gaze restriction, broken pursuit, hypometric saccades, convergent squint	Broken pursuits, Hypometric saccades	Broken pursuits, Hypometric and slow saccades	Normal	Normal

Speech	Dystonic and spastic	Dystonic and spastic	Dystonic and spastic	Dystonic and spastic	Dystonic and spastic	Dystonic and spastic	Dystonic and spastic	Dystonia and spastic

Dystonia*	+	+	+	+	+	+	+	+

Facial twitching	+	+	+	+	+	+	+	+

Tone	Spasticity	Spasticity	Spasticity	Spasticity	Rigidity	Spasticity	Normal	Spasticity

Deep tendon reflexes	Brisk	Brisk	Brisk	Brisk	Normal	Brisk	Brisk	Brisk

Plantar response	Extensor	Extensor	Extensor	Extensor	Flexor	Extensor	Flexor	Extensor

Parkinsonism	–	–	–	+	+	–	–	–

Contractures	+	+	+	+	–	–	–	+

Gait	Dystonic gait Ambulation without support	Dystonic gait Ambulation with support	Dystonic gait Ambulation without support	Dystonic gait Ambulation with support	Dystonic gait Ambulation with support	Dystonic gait Ambulation with support	Dystonic gait Ambulation without support	Dystonic gait. Ambulation with support

Additional findings	Kyphoscoliosis	Kyphoscoliosis	Kyphoscoliosis	Kyphoscoliosis	Kyphoscoliosis	–	Kyphoscoliosis	–

BFMDRS – Movement	NA	66	85	NA	NA	NA	NA	NA

BFMDRS – Disability	NA	11	24	NA	NA	NA	NA	NA

***Imaging***								

Ultrasound abdomen	NA	Normal	Normal	Normal	Normal	Normal	Normal	NA

X-ray spine	NA	NA	Kyphoscoliosis with C5 partial collapse and sclerosis	Kyphoscoliosis	Kyphoscoliosis	NA	NA	NA

Magnetic resonance imaging of the Brain@	BPPAH & WBP	BPPAH & WBP	BPPAH & WBP	BPPAH & WBP, Diffuse cerebral & cerebellar atrophy	BPPAH & WBP, Diffuse cerebral & cerebellar atrophy	BPPAH & WBP	BPPAH & WBP	BPPAH & WBP

***Genetic – GLB1:* (NM_000404.4)**								

Zygosity	CH	CH	CH	CH	CH	HOM	CH	CH

Variant-1 ACMG Criteria Consequence/Novelty	c.1325G>A; p.R442Q P(PS_3_,PM_1__–3_, PP_1–5_) Missense/No	c.1325G>A; p.R442Q P(PS_3_,PM_1–3_,PP_1–5_) Missense/No	c.1325G>A; p.R442Q P(PS_3_,PM_1–3_,PP_1–5_) Missense/No	c.1325G>A; p.R442Q P (PS_3_,PM_1–3_,PP_1–5_) Missense/No	c.1325G>A; p.R442Q P (PS_3_,PM_1–3_,PP_1–5_) Missense/No	c.146G>A; p.R49H P (PS_3_PM_1,2,5_PP_2–5_) Missense/No	c.1325G>A; p.R442Q P(PS_3_,PM_1–3_,PP_1–5_) Missense/No	c.1325G>A; p.R442Q P (PS_3_,PM_1–3_,PP_1–5_) Missense/No

Variant-2 ACMG criteria Consequence/Novelty	c.1022G>T; p.G341V LP (PM_1,2,3_PP_1–4_) Missense/Yes	c.1022G>T; p.G341V LP (PM_1,2,3_PP_1–4_) Missense/Yes	c.246-2A>G LP(PVS_1_PM_2_PP_4,5_) Splice/No	c.202C>T; p.R68W P (PS_3_PM_1,2,5_PP_3–5_) Missense/No	c.1445G>A; p.R482H P (PS_3_PM_1,2,5_,PP_2–5_) Missense/No	Not applicable	c.1370G>A; p.R457Q P (PS_3_, PM_1,2_,PP_2–5_) Missense/No	c.1369C>T; p.R457* P (PVS_1_PM_2,3_PP_4,5_) Stop-gain/No

***Treatment and follow-up***								

Treatment	THP: 12 mg/d LD/CD: 300 mg/d Baclofen: 20 mg/d Clonaz:0.75 mg/d	THP: 12 mg/d LD/CD: 300 mg/d Baclofen: 20 mg/d	THP: 12 mg/d LD/CD: 300 mg/d Baclofen: 20 mg/d	THP: 6 mg/d LD/CD: 400 mg/d Baclofen: 30 mg/d Clonaz: 1 mg/d	LD/CD: 400 mg/d Clonaz: 1 mg/d Quetiapine: 25 mg/d	LD/CD: 400 mg/d Baclofen: 20 mg/d	LD/CD: 300 mg/d Baclofen: 20 mg/d Clonaz: 0.25 mg/d	LD/CD: 400/100 Baclofen: 60 mg/d THP: 6 mg/d

Follow-up	NA	NA	NA	Mild subjective improvement at 9 months	No improvement after 9 months	No improvement after 2 months	Mild subjective improvement at 3 months	No improvement


+: Present; -: Absent; AAO: Age at onset; ACMG: American College of Medical genetics; BFMDRS: Burke-Fahn-Marsden dystonic rating scale; CH: Compound heterozygous; Clonaz: Clonazepam; d: day; DD: Developmental delay; F: Female; FH: Family history; FLAIR: Fluid attenuated inversion recovery; GP: Globus pallidus; HOM: Homozygous; ID: Intellectual disability; LD: Levodopa/carbidopa; LP: Likely pathogenic; M: Male; NA: Not available; SWI: Susceptibility weighted imaging; P: Pathogenic; THP: Trihexyphenidyl; y: Years. *: All patients had generalized dystonia with involvement of oromandibular, lingual, Facial, cervical and all four limbs. In addition, all patients had slow facial twitching or dystonic spasms. All patients had normal sensory examination with no apparent weakness. Cerebellar function could not be tested due to significant dystonia. @: All patients had bilateral symmetrical posterior putaminal atrophy with hyperintensity (BPPAH) on T2 FLAIR and pallidal mineralization on SWI with wish-bone pattern (WBP).

### Clinical history

The median age at onset was 6 years (Range: 3–18 years), with a median age at presentation of 23 years (Range: 12–27 years). All patients presented with abnormal posturing of the whole body, slurred speech, and walking difficulty. In addition, one patient had global developmental delay, and four patients had intellectual disability. None of the patients had any seizures or skeletal abnormalities. A positive family history of recessive inheritance was reported in four patients, and parental consanguinity was noted in one patient.

### Examination

All patients exhibited generalized dystonia, with consistent oromandibular and lingual involvement along with the classical facial dystonia that appears like slow facial twitching, more apparent on speaking (Videos e1, e2, e3, e4). The facial twitching was slower than the usual facial myoclonus, but was more variable and faster to call it a dystonia. It was more akin to facial chorea but is actually slower than the usual facial chorea we see in Huntington’s disease or Neuroacanthocytosis. Also, there was a frequent downward pull of the angle of the mouth, which is typically considered a telltale sign of functional aetiology in patients with hemifacial spasm [4]. Mixed dysarthria in the form of spastic and dystonic speech was noted in all patients. There was spastic component in the form of strained, harsh, strangulated voice quality with slow speech but also occasional variability in articulation with undue pauses at the initiation and in between articulation, and variable but marked overflow and activation of muscles of the jaw and neck on trying to talk that are features of hyperkinetic speech. In addition, parkinsonism was noted in two patients, and corticospinal signs in the form of spasticity, hyperreflexia, and extensor plantar response were seen in six patients. In the presence of dystonia, differentiation between an extensor plantar response and a striatal toe can be challenging. A striatal toe typically occurs spontaneously and in the absence of fanning of the other toes, whereas an extensor plantar response is stimulus-induced and is usually accompanied by fanning of the remaining toes. Similarly, distinguishing between spasticity and rigidity can be challenging in patients with complex movement disorders. However, the presence of velocity-dependent stiffness, predominantly affecting the antigravity muscles, is suggestive of spasticity, whereas velocity-independent stiffness without a predilection for the antigravity muscles is suggestive of rigidity. A detailed cerebellar examination could not be performed owing to severe dystonia. However, the absence of cerebellar ocular signs, ataxic components of speech, and any gait imbalance disproportionate to dystonia suggested that cerebellar involvement was not a prominent feature, even if present. All had dystonic gait, with five patients requiring support to ambulate.

**Video e-1:** **Video of Case-1**. Video demonstrating generalized dystonia involving the face, neck, all four limbs, and trunk. Facial dystonia with slow facial twitching is apparent at rest, which worsens when the patient attempts to speak, along with severe mixed dysarthria.

**Video e-2:** **Video of Case-2**. Video demonstrating generalized dystonia involving the face, torticollis, distal hands and legs, and trunk. Facial dystonia with slow facial twitching and mixed dysarthria is apparent when the patient speaks. The patient can walk independently with a dystonic gait.

**Video e-3:** **Video of Case-3**. Video demonstrating generalized dystonia involving the face, neck, all four limbs, and trunk, with continuous slow facial twitching, and dystonic gait.

**Video e-4:** **Video of Case-5**. Video demonstrating severe cranio-facial dystonia with dysarthria, facial twitching on speaking, and generalized dystonia requiring support to walk.

Among other neurological findings, no obvious cognitive impairment was noted, but a detailed assessment could not be performed due to severe dystonia. Eye movement abnormalities were noted in six patients, including vertical and horizontal gaze restriction, broken pursuit, and/or squint. Additional findings, including contractures, were observed in five cases, and kyphoscoliosis in seven patients. None of the patients had corneal opacity, cataract, demonstrable weakness, sensory impairment, or hepatosplenomegaly.

### Investigations

Blood investigations, which included a hemogram, renal, liver, and thyroid function tests, vitamin B12, homocysteine, folate, ammonia, and lactate levels, as well as tandem mass spectroscopy screening for abnormal metabolites in blood, and routine urine analysis, were normal in all seven patients.

#### Magnetic resonance imaging

MRI brain was available in all patients. The classical bilaterally symmetrical posterior putaminal atrophy with hyperintensity on T2/FLAIR sequencing and globus pallidi interna (GPi) and globus pallidi externa (GPe) hypointensity on SWI imaging with “wishbone” pattern was seen in all patients (Figure 1). Additional cerebral and cerebellar atrophy was noted in three patients. The wishbone, or furcula, is a fused clavicle in birds, characterized by two forked ends joined by a central stem. According to a popular custom, this bone from a cooked turkey is broken by two people holding the ends and making a wish; the person holding the longer fragment is traditionally believed to have their wish granted, hence the term “wishbone”. By analogy, the medial (internal) and lateral (external) segments of the globus pallidus form the two forked ends, while the downward extension of the pallidi represents the stem of the wishbone [5].

#### Other investigations

Electroencephalogram (1/1), visual, auditory, and somatosensory evoked potentials (1/1), nerve conduction studies (3/3), and abdominal ultrasound (6/6) were normal in those for whom it was performed. One patient (Case-1) underwent surface electromyography of the facial muscles around the angle of the mouth and the burst duration of these movements noted to be around 1–2 seconds suggesting dystonic spasms. Three patients had undergone X-ray of the spine and showed kyphoscoliosis in all three, and additional C5 vertebra sclerosis and partial collapse in one patient. None of the three patients demonstrated vertebral beaking—a radiological feature characterized by anterior-inferior, tongue-like protrusions of the vertebral bodies resembling a bird’s beak.

#### Genetic Analysis

Exome sequencing was performed in seven patients, and the remaining patient (Case 2) had undergone Sanger sequencing for the variants identified in his affected sibling (Case 1). All patients had pathogenic or likely pathogenic variants in the *GLB1* gene. Seven patients harbored two variants in a compound heterozygous state, while the remaining patient had a homozygous missense variant (Case 6). Of the total eight variants identified in our cohort, only one variant was novel (c.1022G>T;p Gly341Val), whereas the remaining seven were previously reported (c.1325G>A;p.Arg442Gln, c.146G>A;p.Arg49His, c.246-2A>G, c.202C>T;p.Arg68Trp, c.1445G>A;p.Arg482His, c.1370G>A;p.Arg457Gln, c.1369C>T;p.Arg457Ter). The most frequently observed variant was c.1325G>A;p.Arg442Gln, identified in seven of the eight patients. All patients had biallelic missense variants except for two patients who had a splice acceptor variant (Case-3) and a stop-gain variant (Case-8) in a compound heterozygous state with a missense variant.

##### Treatment and Follow-up

All patients were managed symptomatically with a combination of trihexyphenidyl, levodopa-carbidopa, baclofen, and clonazepam, tailored to dystonia and parkinsonian features. One patient (Case-5) received quetiapine for behavioural symptoms. Follow-up data were available in five patients. Among them, two reported having mild subjective improvement, while the others had no significant clinical response to therapy.

## Discussion

Our cohort delineates the Type-III (adult/chronic) GM1 gangliosidosis phenotype, characterized by an adolescent-to-adult onset and a striking movement disorder profile dominated by generalized dystonia, in which oromandibular and cranio-cervical involvement is early and prominent. Parkinsonian features (bradykinesia/rigidity, hypomimia) and corticospinal signs occurred variably. MRI consistently demonstrated selective basal-ganglia involvement, notably the “wishbone” susceptibility pattern in the globus pallidus with posterior putaminal T2/FLAIR change. Genetic testing frequently identified heterozygous c.1325G>A;p.Arg442Gln, along with another heterozygous variant. Clinically, response to symptomatic therapy was variable but generally mild, mirroring prior literature [1,6,7,8,9]. The uniqueness of this series lies in the convergence of a highly stereotyped oromandibular–cranio-cervical dystonia phenotype with reproducible “wishbone” imaging and a recurrent R442Q background within an Indian centre, features that together strengthen bedside recognition and genotype-phenotype inference in adult GM1 gangliosidosis.

Type-III GM1 gangliosidosis typically manifests as a slowly progressive combined dystonia (often cranio-cervical/oromandibular with generalization) along with corticospinal signs, parkinsonism, ataxia, and dysarthria, but with minimal or no visceromegaly or coarse facies. The facial dyskinesia, especially the lower facial involvement, is an interesting feature of type-III GM1 gangliosidosis and has been described as facial dystonia or facial grimacing in the previous reports [7,10,11]. Although clinically it appears a bit fast for a dystonic movement, one of the patients underwent surface electromyography of the angle of the mouth and had a burst duration of around 1–2 seconds, suggestive of dystonic spasms. Even though cerebellar signs are reported in a few cases of type-III GM1 gangliosidosis [5,12], it usually not frequent in type-3 [7,10,13] compared to the other types [14,15,16]. This may either be due to selective involvement of the brain structures in different subtypes or due to dystonia marring the underlying cerebellar features. Disability accrues over decades and is driven primarily by motor burden; cognition is often normal or only mildly impaired for many years [1,3,6,7]. In contrast, type-I (infantile) begins within the first six months and features rapid neuroregression, visceromegaly, cherry-red spots, dysostosis multiplex, cardiomyopathy, and early lethality [1,17]. Type II (late-infantile/juvenile) presents later with slower neuroregression, atypical seizures, variable skeletal disease without organomegaly [1,17]. These differences parallel higher residual enzyme activity seen in late-onset Type-III disease [1,7].

Radiologically, in Type-III, susceptibility-weighted sequences often reveal the “wishbone” pattern of pallidal mineralization with posterior putaminal involvement, a highly suggestive sign for adult GM1 gangliosidosis when aligned with the clinical picture [5,9]. By comparison, type-I more frequently shows diffuse/delayed myelination, thalamic/brainstem T2 hyperintensities, and early cerebral atrophy. Type-II tends to display patchy white-matter abnormalities and milder atrophy rather than selective pallidal mineralization [1]. Emphasizing this contrast facilitates earlier recognition of adult GM1 gangliosidosis among movement disorder presentations.

Clinically and radiologically, Type-III GM1 gangliosidosis can closely mimic neurodegeneration with brain iron accumulation phenotype. Pantothenate kinase-associated neurodegeneration can have similar phenotype with pallidal mineralization, but in addition has central pallidal hyperintensity resulting in eye-of tiger appearance of T2 MRI sequence. The splitting of pallidal mineralization can mimic mitochondrial membrane protein-associated neurodegeneration; however, it lacks the posterior putaminal atrophy noted in type-III GM1 gangliosidosis. Facial-faucial-finger myoclonus seen in Kufor-Rakeb syndrome can mimic the rapid facial dystonia of type-III GM1 gangliosidosis, but the MRI finding helps in differentiating the two.

Electrophysiological investigations such as EEG, NCS, EMG, and EP are usually normal or nonspecific in Type-III GM1 gangliosidosis. Routine haematology/biochemistry is typically unrevealing. Bone-marrow vacuolated cells or foamy histiocytes, typical of infantile disease are uncommon in late-onset GM1 gangliosidosis, mirroring the paucity of systemic storage signs in adults [1,7]. These negative or nonspecific ancillary tests, when juxtaposed with the characteristic clinical–imaging dyad, help narrow the differential.

Across the GM1 gangliosidosis spectrum, phenotype correlates with residual β-galactosidase activity: infantile forms generally exhibit less than 1% activity, juvenile forms around 1–5%, and adult forms retain higher residual activity, enabling slower substrate accumulation and a later onset [1,2,17,18]. Many late-onset alleles are missense variants that destabilize folding or lysosomal trafficking, or modestly impair catalysis rather than abolish it. Structural studies mapping disease variants onto the (β/α)8 TIM-barrel catalytic domain and adjacent β-domains reveal that changes outside catalytic residues often preserve measurable activity and partial lysosomal targeting—molecular features congruent with the Type-III phenotype [2,17,18].

The c.1325G>A;p.Arg442Gln or R442Q variant is repeatedly linked with late-onset/Type-III GM1 gangliosidosis subtype (Table 2) [2,18,19,20,21]. Its absence from a large Indian multicenter *GLB1* survey suggests regional heterogeneity of variant spectra [22]. In a well-characterized adult compound-heterozygote (p.T329A/p.R442Q), fibroblast studies demonstrated residual β-galactosidase activity that increased with galactose, supporting a misfolding/stability defect rather than catalytic ablation [23]. Longitudinal experience indicates that substrate-reduction therapy with miglustat may stabilize or improve disease in juvenile/adult GM1 gangliosidosis—including this lineage—underscoring that R442Q aligns with residual-activity, potentially therapy-amenable biology. Collectively, R442Q sits among late-onset alleles with plausible chaperone/SRT responsiveness.

**Table 2:** Studies reporting cases with the variant c.1325G>A;p.R442Q.


STUDY	AGE/AAO/SEX/TYPE*	PRESENTATION	INVESTIGATIONS

**Caciotti et al, 2005**	27/5/NA/Adult	Short stature, ataxia, speech deterioration, bilateral clubfoot, corneal opacity, normal IQ	Other variant: c.985A>G;p.T329A MRI: Mild hyperintensity in the posterior periventricular white matter and mild alteration in the lenticular nuclei GLB1 activity: 4.1 in leukocytes

**Hofer et al, 2009**	30/11/NA/Juvenile	CNS and skeletal involvement with no cherry red spot, cardiac involvement, or hepatosplenomegaly	Other variant: c.986C>T;p.T329L MRI: NA GLB1: NA

**Hofer et al, 2010**	16/4/NA/Adult	CNS and skeletal involvement with no cherry red spot, cardiac involvement, or hepatosplenomegaly	Other variant: c.1077delA;p.K359Kfs*23 MRI: NA GLB1 activity: 5–15 in leucocytes

**Caciotti et al, 2011**	9/4/Female/Juvenile	Dysarthria, tremor, ataxia, borderline IQ, dystonia, stiffness. No eye abnormality/organomegaly	Other variant: c.275G>A;p.W92* MRI: NA GLB1 activity: 2 in leucocytes; 7.5 in fibroblasts

**Kumar et al, 2016**	20/Infancy/Female/NA	Spastic limbs, limb dystonia, developmental delay, marked speech disturbance, and unable to mobilise.	Other variant: c.553–2A>G

	18/Infancy/Female/NA	Spastic limbs, limb dystonia, developmental delay, marked speech disturbance, unable to mobilise	

	12/Infancy/Male/NA	Spastic gait, limb dystonia, developmental delay, drooling of saliva. No organomegaly or skeletal manifestations	MRI: globus pallid hypointensities were evident on repeat evaluation GLB1 activity: 1.6 in leucocytes


*: Reported type in the original article. AAO: Age at onset; CNS: Central nervous system; IQ: Intelligent quotient; MRI: Magnetic resonance imaging; NA: Not available.

Currently, no disease-modifying therapies are approved for GM1 gangliosidosis. Care remains multidisciplinary and supportive symptomatic therapy. All patients in our cohort were managed with symptomatic therapy with variable subjective improvement. However, promising therapies are in development, particularly for late-onset forms. Approaches under investigation include gene therapy, substrate reduction therapy, pharmacological chaperones to stabilize misfolded enzyme proteins, and hematopoietic stem cell transplantation. The presence of residual enzyme activity and slower disease progression in Type-III GM1 gangliosidosis makes these patients ideal candidates for future interventions. Substrate-reduction therapy with miglustat has shown stabilization or improvement in the juvenile–adult GM1 gangliosidosis series [17]. Pharmacologic chaperoning (for example, galactose and iminosugar compounds such as NOEV) can augment residual β-galactosidase activity in an allele-specific fashion, including R442Q compound-heterozygous contexts. Gene-therapy programs using AAV-mediated GLB1 delivery are progressing in pediatric cohorts; although adult/Type-III data remain limited, the shared pathobiology supports future applicability as delivery and dosing strategies evolve. Due to the lack of established benefit of deep-brain stimulation (DBS) in GM1 gangliosidosis and complex dystonia manifestation, we did not offer DBS for any of the patients.

Our study contributes valuable real-world data on a rare condition, particularly from an underrepresented region. It highlights a consistent clinical and imaging phenotype, reinforcing the significance of recurrent GLB1 mutations in the Indian context.

Limitations include the retrospective nature of data collection and the limited availability of long-term follow-up.

## Conclusion

This case series highlights the distinct clinical, radiological, and genetic features of late-onset GM1 gangliosidosis in an Indian cohort. Our findings reinforce that Type-III GM1 gangliosidosis should be considered a key differential diagnosis in young individuals presenting with progressive generalized dystonia, particularly when accompanied by predominant oromandibular and facial involvement, and corticospinal signs. The consistent presence of the “wishbone pattern” on MRI and the recurrent detection of the p.Arg442Gln *GLB1* variant further strengthen the diagnostic framework.
